# Evaluating the relationship between the proportion of X-chromosome deletions and clinical manifestations in children with turner syndrome

**DOI:** 10.3389/fendo.2024.1324160

**Published:** 2024-02-28

**Authors:** Gaowei Wang, Xiaojing Liu, Meiye Wang, Jin Wang, Zhenhua Zhang, Karel Allegaert, Daoqi Mei, Yaodong Zhang, Shuying Luo, Yang Fang, Dongxiao Li, Yongxing Chen, Haiyan Wei

**Affiliations:** ^1^ Henan Key Laboratory of Children’s Genetics and Metabolic Diseases, Henan Children’s Neurodevelopment Engineering Research Center, Children’s Hospital Affiliated to Zhengzhou University, Henan Children’s Hospital, Zhengzhou Children’s Hospital, Zhengzhou, China; ^2^ Department of Endocrinology, Genetics and Metabolism, Children’s Hospital Affiliated to Zhengzhou University, Henan Children’s Hospital, Zhengzhou Children’s Hospital, Zhengzhou, China; ^3^ Department of Development and Regeneration, KU Leuven, Leuven, Belgium; ^4^ Department of Pharmaceutical and Pharmacological Sciences, KU Leuven, Leuven, Belgium; ^5^ Department of Hospital Pharmacy, Erasmus MC, Rotterdam, Netherlands; ^6^ Department of Neurology, Children’s Hospital Affiliated to Zhengzhou University, Henan Provincial Children’s Hospital, Zhengzhou Children’s Hospital, Zhengzhou, Henan, China; ^7^ Department of Laboratory Medicine, Third Affiliated Hospital of Zhengzhou University, Zhengzhou, Henan, China

**Keywords:** turner syndrome, X-chromosome, short stature, mosaicism, phenotype

## Abstract

**Purpose:**

Analyze the relationship between changes in the proportion of X-chromosome deletions and clinical manifestations in children with Turner syndrome (TS).

**Methods:**

X-chromosome number abnormalities in 8,635 children with growth retardation were identified using fluorescence *in situ* hybridization (FISH). Meanwhile, the relationship between the proportion of X-chromosome deletions and the clinical manifestations of TS, such as face and body phenotype, cardiovascular, renal, and other comorbidities in children with TS was analyzed.

**Results:**

A total of 389 children had X-chromosome number abnormalities, with an average age at diagnosis of 9.2 years. There was a significant increase in diagnoses around the ages of 3 and 7 years and highest number of diagnoses at 10 years of age. 130 with XO (complete loss of an X-chromosome), 205 with XO/XX, 8 with XO/XXX, 23 with XO/XX/XXX, 19 with XO/XY, and 4 with XO/XY/XYY. Body and facial phenotypes increased with higher mosaicism proportions, with a relatively high correlation shown with Pearson correlation analysis (*r* = 0.26, *p* = 1.7e-06). The incidence of congenital heart malformations was 25.56%, mainly involving a bicuspid aortic valve, and were more common in patients who had complete loss of an X-chromosome. However, this relationship was not present for renal disease (*p* = 0.26), central nervous system, thyroid, or liver disease.

**Conclusion:**

The mosaicism (XO/XX) is the most common karyotype of TS in screened cases. The phenotypes in children with TS may increase with the proportion of X-chromosome deletions, but the renal disease and comorbidities did not show the same characteristics.

## 1 Introduction

Sex chromosome abnormalities (SCAs) are the most common chromosomal disorders, with an incidence of 1 in 400 newborns. Turner syndrome (TS) is one of the most well-known SCA syndromes (1). TS is characterized by the absence of one or part of an X-chromosome. Comprehensive data from a number of European and Asian countries show that the estimated prevalence in newborns is approximately 64 per 100 000, and the number of females actually diagnosed was from 17 to 35 per 100 000 (2). The clinical characteristics of TS have been well-described, and include short stature, webbed neck, characteristic facial features, cardiac defects, renal abnormalities, endocrine problems, hearing problems, and infertility (3). TS also appears to involve the eyes and nose (4). Women with TS may present with different karyotypes. Approximately 40%–50% of women with TS present with the 45,X karyotype, 15%–25% with 45,X/46,XX, 3% with 45,X/47,XXX and 45,X/46,XX/47,XXX, 10% with 45,X/46,XY, and the rest with rarer structural anomalies (3). Recent studies have shown that the proportion of TS patients diagnosed with a mosaicism (45,X/46,XX, 45,X/47,XXX, 45,X/46,XX/47,XXX) is increasing (1, 5). Traditional techniques (Chromosome karyotype analysis) for defining the distribution of TS karyotypes have been challenged by the utilization of fluorescence *in situ* hybridization (FISH) and single nucleotide polymorphisms (SNP) (6). Mosaic TS has a reduced penetrance in the adult population, indicating that more mosaicisms are less likely to be detected (7).

As mentioned in a recent review, many questions about TS remain unanswered, such as the genetic mechanisms behind TS, how to improve its diagnosis, the optimal time of hormone replacement therapy, the optimal dose and route to be used for fertility treatment at different ages, and care settings (8). The relationship between TS karyotype and clinical phenotype is not well understood due to its wide age distribution, variability in the definition of its clinical phenotype, and differences in the X mosaicism of different tissues (2, 3).

FISH is faster and more sensitive than karyotyping, permitting the identification of quantitative changes in the proportion of abnormal cells with genetic mosaicism. We therefore used FISH to detect the number of sex chromosomes in peripheral blood leukocytes of patients with TS and compared it with some known TS phenotypes in order to better understand the relationship between the proportion of X-chromosome deletions and phenotype.

Our study analyzed children who presented for evaluation of growth retardation, the clinical manifestations of short stature, and/or other physical features associated with TS. Individuals with an X-chromosome (XO) deletion were identified and mosaicisms (XO/XX, XO/XXX, XO/XX/XXX, XO/XY, XO/XY/XYY) were identified. Our team used information from outpatient and inpatient medical records to collect clinical information about each patient, allowing for the clinical characterization of TS during childhood and adolescence. We sought to identify the relationship between clinical phenotype and the proportion of X-chromosome deletions in children with TS.

## 2 Materials and methods

### 2.1 Patient cohort

A retrospective analysis of 8,635 children who presented with growth retardation, clinical manifestations consistent with TS, or a diagnosis of short stature to Henan Children’s Hospital from January 2010 to December 2022 was performed. TS patients (n = 389) with complete or partial deletion of the X-chromosome number were included in this study. All children are female by gender, aged from 0 to 18 years. All hospitalized children had an abdominal ultrasound and some with signs or symptoms of TS had ultrasound of the relevant organs. We reviewed consultation records to extract genetic results, face and body phenotype and cardiovascular, renal, hepatic, abdominal, gastrointestinal, and thyroid disorders. A systematic analysis of the genetic characteristics, clinical features, and age distribution of TS was performed to determine if there was a correlation between those characteristics and karyotype.

This study was approved by the Ethics Committee of Henan Children’s Hospital (2022-K-L020). The study was performed in accordance with the relevant guidelines and regulations. Informed consent was waived by the Ethics Committee of Henan Children’s Hospital.

### 2.2 FISH analysis

Blood samples were collected from patients under sterile conditions using heparin as an anticoagulant. Sex chromosome detection was performed using the CSPX/CSPY sex chromosome centromere probe (Cytocell, UK), and hybridization was performed with the TDH-500 *in situ* hybridization instrument (Thermo Brite). All procedures followed the FISH protocol, including leukocyte extraction, slide preparation, probe application, overnight hybridization, elution, staining, and microscopic examination. Multi-color fluorescence images were captured and analyzed using a fluorescence microscope (BX51 OLYMPUS). A total of 100 cells from each specimen were counted under the microscope. Cells with X-chromosome number abnormalities (XO) at the lowest level of mosaicism were excluded (5% with 99% confidence) (9). Each sample was reviewed by two examiners.

### 2.3 Statistics and data analysis

We systematically analyzed genetic characteristics, clinical presentation, age at diagnosis, height and weight, and the proportion of X-chromosome deletions. Bar charts and line graphs were produced with OriginPro 2016. Associations between the proportion of X-chromosome deletions and clinical phenotype and age at diagnosis were analyzed. A total of 334 children with TS had different proportions of X-chromosome mosaicism. A summary classification of age and facial and body phenotypes was performed. Facial and body phenotypes were counted (e.g. a child with a low hairline, cubitus valgus, shield chest, sinusitis, and otitis media was counted as 5). Correlations were analyzed using pearson correlation analysis with the R package “ggpubr” and a correlation range of [−1,1]. A Kruskal-Wallis analysis of concomitant renal disease in children with TS and different mosaicism proportions was also performed using R, with p-values less than 0.05 indicating a significant difference.

## 3 Results

### 3.1 Types of SCAs in TS

**Figure 1:**
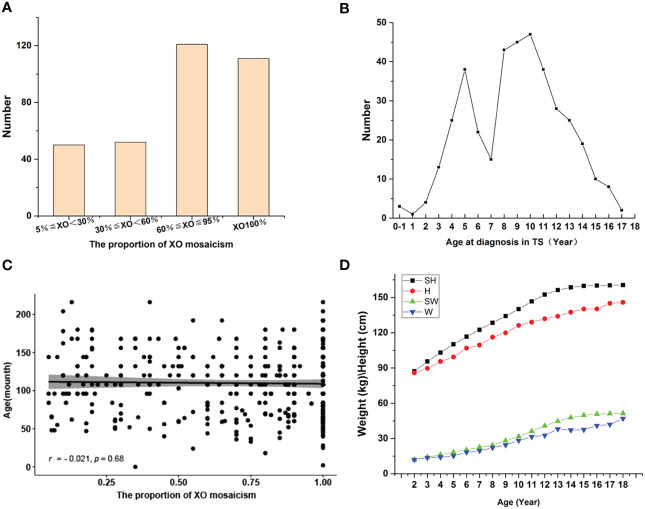
**(A)** Distribution of XO mosaicism. Patients were divided into four groups: XO<30% (50), 30%≤XO<60% (52), 60%≤XO ≤ 95% (121), XO100% (111). **(B)** Age distribution of children diagnosed with TS. **(C)** Relationship between age at TS diagnosis and the proportion of XO mosaicism. The X-axis represents the change in the proportion of XO mosaicism, from 0.05 to 1. The Y-axis indicates the age distribution of each child at different proportions of XO mosaicism. r-values indicate correlation range [−1,1]. **(D)** Changes in the height and weight of children diagnosed with TS vs. normal controls. Abbreviations: standard height (SH), height of those diagnosed with TS (H), standard weight (SW), weight of those diagnosed with TS (W).

A total of 389 children had X-chromosome abnormalities and clinical features consistent with TS. Of these, 130 children had an XO (XO 100%), 205 cases XO/XX, 8 XO/XXX, 23 XO/XX/XXX, 19 XO/XY, and 4 XO/XY/XYY (**Table 1**). 334 children with complete outpatient and inpatient medical records were artificially divided into four groups of 5%≤XO<30% (50 cases), 30%≤XO<60% (52 cases), 60%≤XO ≤ 95% (121 cases), and XO 100% (111 cases) (**Figure 1A**).

**Table 1:** Types and distribution of SCAs in 389 cases TS.

Types of SCAs in TS	Number	Percentage
**XO**	130	33.42%
**XO/XX**	205	52.70%
**XO/XXX**	8	2.06%
**XO/XX/XXX**	23	5.91%
**XO/XY**	19	4.88%
**XO/XY/XYY**	4	1.03%

### 3.2 Age at TS diagnosis

Age at TS diagnosis ranged from birth to 18 years, with average age at diagnosis of 9.2 years. Three cases were diagnosed during the neonatal/infancy period (0-1 month to <1 years), 253 cases during childhood (1 to <11 years), and 133 cases during adolescence (11–18 years) (**Table 2**). Highest number of diagnoses at 10 years of age and there was a significant increase in diagnoses around the ages of 3 and 7 years (**Figure 1B**). We further analyzed the relationship between age at TS diagnosis and the proportion of X mosaicism. Our correlation was calculated as *r* = −0.021, *p* = 0.68 (**Figure 1C**), indicating that age at diagnosis was not associated with the proportion of X mosaicism.

**Table 2:** Number of confirmed diagnoses at different growth periods with 389 cases TS.

Age	Case number	Mean age/year
Neonatal/Infancy (0–1 month to <1 years)	3	0.3
Childhood (1 to <11 years)	253	7.28
Adolescence (11–18 years)	133	12.92
Total	389	9.2

### 3.3 Clinical features of TS

The majority of the children with TS had short stature (95.81%). Complete outpatient and inpatient medical records were analyzed and classified according to clinical phenotype. A comparison of standard heights and weights at different ages with the corresponding heights and weights in this study found that the height gap became more pronounced after the age of 10 years. (**Figure 1D**). All children diagnosed with TS underwent Doppler ultrasound examinations of their ovaries. These examinations showed 108 cases with poorly shown ovaries bilaterally, 189 cases with the confirmed presence of one or both ovaries, and 37 cases with an unilateral ovary.

#### 3.3.1 Face and body phenotypes

The combined face and body phenotypes of 334 children with TS were analyzed. The most common abnormalities were in the skeletal system, including cubitus valgus, widely spaced nipples, and a shield chest, followed by skin nevi or spots, craniofacial abnormalities, webbed neck, and ear, eye, and teeth abnormalities (**Table 3**). The most commonly observed abnormality of the craniofacial region was a low hairline. The screening process for high palatal arches and small mandibles was not very well reported as only a few of them were described in diagnostics. Refractive error, epicanthi, and ptosis were the most commonly observed eye abnormalities. Otitis media was the most prevalent ear abnormality and there was a high prevalence of nasal diseases in our cohort, primarily sinusitis and rhinitis. Facial and body nevi were common skin disorders in this population with our study, in addition to some children with skin diseases such as dermatitis and eczema. Skeletal disorders were the most prevalent in the children with TS. Some of the children had a combination of multiple skeletal deformities, mainly cubitus valgus, widely spaced nipples, shield chest, abnormal metacarpals, and short neck. Additionally, some children present with abnormality of the lower limb, scoliosis or congenital hip dislocation.

**Table 3:** Phenotype of the face and body in 334 cases TS.

Phenotype	Number	%
**Craniofacial (**low hairline)	**97**	**29.04**
low hairline	97	
**Teeth**	**22**	**6.59**
Carious teeth	10	
Dentifacial deformity	6	
Hypoplasia of dental enamel	4	
Persistence of primary teeth	3	
Dentinogenesis imperfecta, Premature loss of primary teeth, gingival atrophy	3	
**Eye**	**39**	**11.68**
Refractive error	16	
Epicanthus	13	
Ptosis	7	
Conjunctivitis	6	
Nystagmus	3	
Strabismus	3	
Hypertelorism	2	
Amblyopia	1	
Hypermetropia	1	
Astigmatism	1	
**Ear**	**73**	**21.86**
Otitis media	56	
Ceruminal impaction	14	
External ear malformation	5	
Cholesteatoma of the middle ear canal	1	
**Nose**	**194**	**58.03**
Sinusitis	174	
Enlargement of the inferior turbinate	60	
Rhinitis	10	
**Skin**	**111**	**33.23**
Facial and body nevi	94	
Cafe-au-lait spots	16	
Leucoderma	3	
**Webbed neck**	**69**	**20.66**
**Skeletal system**	**206**	**61.68**
Cubitus valgus	127	
Widely spaced nipples	97	
Shield chest	73	
Short fourth or fifth metacarpals	64	
Short neck	36	
Abnormality of the lower limb	13	
Scoliosis, congenital hip dislocation	11	
Lumbar kyphosis, chondromatosis, ect	9	

**Figure 2:**
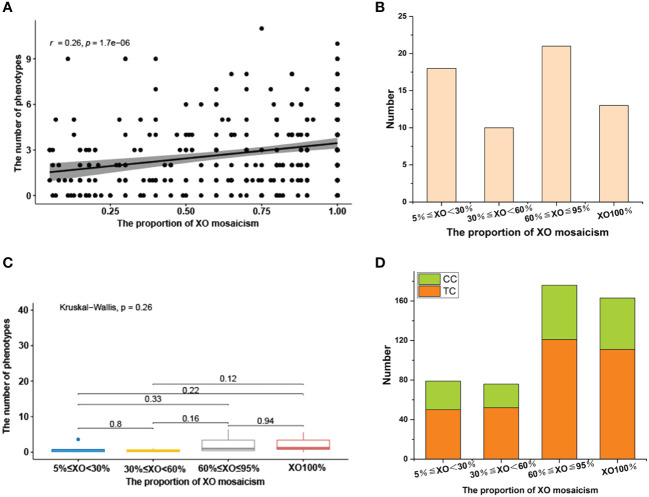
**(A)** Relationship between face and body phenotypes and the proportion of XO mosaicism. The X-axis represents change in the proportion of XO mosaicism, from 0.05 to 1. Y-axis indicates the number of phenotypes involved per child at different proportions of XO mosaicism. r-values indicate correlation range [−1,1]. **(B)** Distribution of sex chromosomal abnormalities with different proportions in 62 children without facial or body phenotypes. 5%≤XO<30% (18), 30%≤XO<60% (10), 60%≤XO ≤ 95% (21), XO100% (13). **(C)** Differences in the prevalence of renal disease across different proportions of XO mosaicism. The X-axis represents change in the proportion of XO mosaicism. The Y-axis represents phenotype count. p-values less than 0.05 indicate a significant difference. **(D)** Distribution of SCAs with different proportions of XO mosaicism in 160 children with brain, thyroid, and gastrointestinal abnormalities. XO<30% (29/50), 30%≤XO<60% (24/52), 60%≤XO ≤ 95% (55/121), XO100% (52/111), Comorbidities case (CC), Total case (TC).

Our correlation analysis of facial and body phenotype and XO mosaicism proportion indicated a positive association, with a correlation coefficient (*r*) of 0.26 (degrees of freedom (df) = 332), *p* = 1.7e-06. (**Figure 2A**). It should be noted that 62 children with different types of sex chromosome number abnormalities did not have any other clinical manifestations of TS except for short stature. There was no significant correlation or trend in the distribution of different X mosaicisms in children with TS without facial or body phenotypes (**Figure 2B**).

#### 3.3.2 Concomitant diseases with TS

Individuals with TS may suffer from cardiovascular, renal, hepatic, abdominal, gastrointestinal, central nervous system (CNS), and thyroid disorders, leading to long-term distress (**Table 4**). We identified 334 children with TS who had such conditions and analyzed their genetic characteristics.

**Table 4:** Phenotype of the cardiovascular system, renal, thyroid, ect.

Phenotype	Number(334 cases in total)
**Cardiovascular system**	**82**
Aorta	
Bicuspid aortic valve	23
Coarctation of the aorta	10
Widening of aortic internal diameter	6
Patent ductus arteriosus	4
Mitral	
Mitral regurgitation	10
Mitral stenosis	4
Left ventricular false tendon	33
Patent foramen ovale	10
Persistent left superior vena cava	6
Coronary artery anomalies	4
Ventricular septal defect	1
Atrioventricular septal defect	3
left ventricular wall thickening	3
Hypertension and dyslipidaemia	7
**Renal**	**70 **
Congenital malformation of the kidney	
Renal pelvis separation	41
Horseshoe kidney	24
Hydronephrosis	6
Duplication of renal	3
Polycystic kidney	1
Other urinary system disease	
Abnormality of renal calyx morphology	1
Hematuria	2
Left renal vein compression syndrome	2
Nephrolithiasis	1
Uroparesis	1
Kidney injury	1
**Central Nervous System**	**41**
Arachnoid cyst in the occipital region	11
Widened subarachnoid space in the occipital region	12
Rathke’s cyst	4
Pineal cyst	4
Neuropathic headache	4
Brain injury	3
Hydrocephalus, Anoxic-ischaemic encephalopathy, Encephalitis, ect	6
**Thyroid disease**	**28 **
Hypothyroidism	6
Hyperthyroidism	2
Thyroiditis	10
Abnormal thyroid function	4
Thyroid cysts	8
Abdominal, Gastrointestinal and Liver disease	
Abdominal	**40 **
Abdominal pain	22
Abdominal lymph nodes	20
Gastrointestinal	**27 **
Gastroenteritis	25
Rectal polyposis, Bowel adhesions, Bowel obstruction, Intussusception	8
**Liver disease**	**25 **
Hepatomegaly	5
Liver function abnormalities	7
Fatty liver	11
Abnormalities of the portal vein	2

##### 3.3.2.1 Cardiovascular system

Girls with TS face a lifelong heavy burden of congenital and acquired cardiovascular diseases, with increased mortality and morbidity. A total of 180 children in our cohort underwent cardiac ultrasound Doppler screening, yielding a screening rate of 53.89% (180/334). Of these, 82 children had cardiovascular disease, 78 (43.33%, 78/180) had congenital heart disease, 6 had hypertension and dyslipidaemia, and overall, the incidence of cardiovascular disease is 43.33% (82/180). The Doppler ultrasound show more frequent and more severe manifestations of aortic malformations (31) (**Table 4**). Other malformations included a patent ductus arteriosus (4), mitral valve (15) and coronary artery anomalies (4), left ventricular pseudotendon (33), patent foramen ovale (10), persistent left superior vena cava (6), anomalous coronary arteries or veins (5), ventricular septal defects (1), atrioventricular septal defects (3), left ventricular wall thickening (3), and hypertension and dyslipidemia (7). Mitral regurgitation, a left ventricular false tendon, and a patent foramen ovale had a prevalence in normal subjects. However, when these common anomalies in normal subjects were excluded, these more severe anomalies, such as aortic malformations, which were present in only 46 (25.56%) cases. The detailed distribution of these anomalies are shown in **Table 5A** and **Table 5B**. Aortic anomalies mainly involved a bicuspid aortic valve (BAV). There was a higher incidence of congenital heart anomalies in TS patients who had a complete loss of an X-chromosome which accounts for 32.79% of TS with congenital heart anomalies in our study, and among those who had a high prevalence of TS phenotypes.

**Table 5A:** Distribution characteristics of cardiovascular disease at different X mosaicism proportions.

X proportion	Bicuspid aortic valve	Coarctation of the aorta	Widening of aortic internal diameter	Patent ductus arteriosus	Mitral stenosis	Persistent left superior vena cava	Anomalous coronary arteries or veins
**XO≦30%**	3	1	1	N	N	1	1
**30%<XO≦60%**	5	N	N	N	N	N	1
**60%<XO≦95%**	5	2	3	3	2	1	N
**XO100%**	10	8	3	1	2	4	2
**Total**	23	11	6	4	4	6	4

**Table 5B:** Distribution characteristics of cardiovascular disease at different X mosaicism proportions.

X proportion	Ventricular septal defect	Atrioventricular septal defect	left ventricular wall thickening	Hypertension and dyslipidaemia	Total case	Cardiovascular case (%)	Phenotype number (%)
**XO≦30%**	N	N	1	N	**30**	**5 (16.66)**	**8 (26.66)**
**30%<XO≦60%**	N	N	N	2	**32**	**4 (12.50)**	**7(21.86)**
**60%<XO≦95%**	1	3	N	1	**57**	**17(29.82)**	**21(36.84)**
**XO100%**	N	N	2	4	**61**	**20 (32.79)**	**36(59.02)**
**Total**	1	3	3	7	**180**	**46 (25.56)**	**-**

##### 3.3.2.2 Renal disease

A total of 329 children with TS in our cohort underwent renal ultrasound Doppler screening, yielding a screening rate of 98.50% (329/334). Of these, 70 (21.27%, 70/329) had some type of renal disease, including renal pelvis separation (41), horseshoe kidney (24), hydronephrosis (6), duplicated kidney (3), and polycystic kidney (1). Other detected urologic disorders included nephrolithiasis (1), abnormal renal calyx morphology (1), hematuria (2), uroparesis (1), kidney impairment (1), and left renal vein compression syndrome (2) (**Table 4**). A Kruskal-Wallis analysis evaluated if there were significant differences in the incidence of renal disease and different X mosaicism proportions (**Table 6**). No significant difference was observed, with an overall p-value of 0.26 and all p-values greater than 0.05 for comparisons between individual mosaicism proportions (**Figure 2C**).

**Table 6:** Distribution characteristics of renal disease at different X mosaicism proportions.

X proportion	Hydronephrosis	Renal pelvis separation	Horseshoe kidney	Duplication of renal	Polycystic kidney	Other urinary system disease	Total case	Renal case (%)	Phenotype number (%)
**XO<30%**	**2**	**9**	**1**	N	N	**2**	**50**	**10 (20.00)**	**13 (26.00)**
**30%≦XO<60%**	**1**	**2**	**3**	N	N	**1**	**51**	**5 (9.80)**	**7 (13.73)**
**60%≦XO≦95%**	**1**	**16**	**10**	**1**	**1**	**4**	**119**	**29 (24.37)**	**33 (27.73)**
**XO100%**	**2**	**14**	**10**	**2**	N	**4**	**110**	**25 (22.72)**	**32 (29.09)**
**Total**	**6**	**41**	**24**	**3**	**1**	**11**	**329**	**70 (21.27)**	**-**

##### 3.3.2.3 Central nervous system, thyroid, abdominal, gastrointestinal, and liver disease

Patients with TS had an increased incidence of several other systemic disorders (**Table 4**). CNS involvement was identified in 41 children, including an arachnoid cyst in the occipital region (11) and a widened subarachnoid space in the occipital region (12). Thyroid hormone dosage measurement is necessary for patients diagnosed with TS,whose were checked at the first visit and then were reviewed with regular treatment. Twenty-eight children had thyroid disorders and were detected during treatment, including hypothyroidism (6), hyperthyroidism (2), thyroiditis (10), and thyroid cysts (8), abnormal thyroid function (4). Gastrointestinal disorders included gastroenteritis (25), rectal polyposis (4), bowel adhesions (2), bowel obstruction (1), and intussusception (1). Abdominal pain (22) and abdominal lymph nodes (20) were also identified (**Table 2**). Liver lesions were found in 25 children with TS, including fatty liver (11), liver function abnormalities (7), hepatomegaly (5), and portal vein abnormalities (2). A total of 160 children (47.90%) had some systemic abnormality. There was no significant difference in the distribution of the above symptoms and X mosaicism proportion (**Figure 2D**).

## 4 Discussion

For individuals with TS, it is crucial to identify the early features of TS to make a clinical diagnosis and initiate early treatment. Previous studies on TS primarily focused on adults, or included a broader age distribution and more systematically described the clinical presentation of patients with TS during different growth periods (7, 8, 10, 11). We combined the past 13 years of testing records for individuals under the age of 18 years. A total of 8,692 cases were included in our cohort, of which 389 cases with X-chromosome number abnormalities (detection rate of 4.48%) were analyzed. Our team systematically classified and quantified the clinical manifestations of 334 of these patients who had comprehensive outpatient and inpatient medical records, permitting analysis of the distribution of SCAs in children with TS and the relationship between these abnormalities, age at diagnosis, and clinical presentation.

The average age at diagnosis was 9.2 years, in accordance with previous studies on the average age of children with TS diagnosed between 0 and 18 years (12, 13). Our study found that most children were diagnosed at age 10, with an increased number of diagnosis around ages of 3 and 7. These two periods coincided with the age at which children started kindergarten and primary school in China. It is likely that parents observed that the height of their child was lower than that of its peers, leading to parental concern and subsequent medical evaluations. It is therefore important to perform comprehensive multidisciplinary examinations with specialized physicians during the initial examination of school-age children to facilitate the early diagnosis and treatment of such abnormalities. Approximately two-thirds (66.08%) of the children with TS were diagnosed during childhood, with an average age of 7.2 years. The remaining cases were mostly diagnosed during adolescence, with an average age of 12.1 years.

When TS is diagnosed during childhood, the optimal age for growth hormone treatment (GH) is 4–6 years (3). Our data suggests that children diagnosed during kindergarten were more likely to receive timely and comprehensive treatment. However, the mean age of diagnosis during childhood in our study was 7.2 years, suggesting that many children are diagnosed after entering primary school and therefore miss the optimal time for GH treatment. The number of TS diagnoses during adolescence decreased with increasing age, with a mean age of 12.1 years. Early GH therapy allowed TS patients to achieve normal heights during childhood and adulthood, thereby minimizing physical restrictions and allowing puberty to begin at a similar age to peers. There was a significant difference in height after puberty initiation in patients diagnosed during adolescence because estrogen replacement therapy was not started before puberty (3). TS diagnosed at an age older than 12 years is a medical challenge and a persistent problem. New strategies for early detection would permit the start of specialized therapies and multidisciplinary interventions for better individualized treatment (14). Our data indicates that age at diagnosis is not associated with the proportion of X-chromosome number abnormalities, with a correlation coefficient(*r*) of −0.021 (degrees of freedom (df) = 387), *p* = 0.68. This suggests that TS with different types of X-chromosome abnormalities can be identified at any age and that early and comprehensive screening can better detect disorders in sex development caused by SCAs, permitting improved clinical management and outcome.

### 4.1 Changes in the distribution of SCAs in TS

Data from the 2016 Cincinnati International Turner Syndrome Conference found that about 40%–50% of women with TS had a 45,X, karyotype (3). In contrast, our work found mosaicisms (XO/XX, XO/XXX, XO/XX/XXX, XO/XY) in 255 of 389 children (65.55%), with an increasing rate of mosaicism over time. This disagreement may be attributed to several factors. First, our study included short stature as a broader inclusion criterion in addition to the more obvious characteristics of TS, resulting in a wider range of patients being analyzed. Among the patients with more complete case records, 62 presented with only short stature, without a more pronounced TS phenotype and associated conditions. Second, the CSPX/CSPY sex chromosome centromere probe permitted 100 cells to be counted, permitting low percentage X mosaicisms to be detected accurately and with greater sensitivity. These patients had no karyotype abnormalities but FISH testing detected low proportions (about 20%–30%) of X-chromosome mosaicism. This suggests that the combined application of multiple techniques provides better support for clinical diagnosis and research. Urbach et al. found that approximately 99% of fetuses with karyotype 45,XO had spontaneous abortions during early or mid-pregnancy, whereas those with fetuses with 45,XO/46 and XX mosaicism were more likely to survive (15). This further suggests that there may be more children with X-chromosome mosaicism whose clinical presentation is unrecognized.

We therefore believe that sex chromosome testing should be performed to determine whether a patient with a significantly smaller stature than children of the same age has a sex chromosome abnormality. We also believe that the existing descriptions of the distribution of sex chromosomal abnormalities in children with TS based on karyotyping techniques ignore patients with a low percentage of mosaicisms, and that the prevalence of mosaicisms in TS may be underestimated overall. FISH is fast, sensitive, and convenient, but is not able to detect structural abnormalities.

In addition to the study of children with TS mentioned above, four children with an X mosaicism less than 5% were identified during practical testing, two of which had the distinct facial and body features of TS in addition to comorbidities such as hydronephrosis, otitis media, and abdominal pain. The combined use karyotyping along with multiple analysis techniques such as whole-exome sequencing (WES), whole genome copy number variant sequencing (CNV-seq), and FISH in different tissues can provide a more accurate TS diagnosis.

### 4.2 Relationship between X mosaicism proportion and face and body phenotype

The wide clinical spectrum of TS includes a range of classical appearances that varies between individuals, with craniofacial, dental, eye, ear, nose, skin, and skeletal system abnormalities more pronounced and widely discussed (3, 8, 10, 12, 13, 16). Our Pearson correlation analysis showed a relatively high correlation (*r* = 0.26) between such abnormalities and mosaicism (*p* = 1.7e-06). However, not all patients with a low proportion of mosaicisms had fewer clinical phenotypes, and *vice versa*. There were still 62 children in this study who had different types of sex chromosome number abnormalities but did not have any of the above clinical manifestations except for short stature, including more than half of the children who had a high proportion of X mosaicism or complete loss of an X-chromosome. Overall, there was no significant correlation or trend in the distribution of different X mosaicism proportions in children with TS without facial and body phenotypes. This indicates that different proportion of X-chromosome deletions do not explain the variability in the facial and body phenotypes in some patients with TS. As described in previous studies, the consequences of X-chromosome deletions are highly variable, ranging (at the extremes) from intrauterine death to normal health (17). Further follow-up investigations of this subset of TS patients would be more meaningful.

Skeletal system disorders had the highest prevalence in our cohort, with 61.68% having some abnormality beyond delayed bone age. With respect to nasal disorders, patients in our cohort had a high prevalence of sinusitis (52.10%), but some pathologies such as otitis media were not as well studied. Otitis media has been reported to be prevalent in about 30% of patients with TS (3), but only accounted for 16.77% of the patients in our study. Enhanced screening for complications in children with TS at the time of clinical diagnosis and subsequent treatment is necessary. Data from the present work supports our assertion that children with a high proportion of X mosaicisms or the complete loss of an X-chromosome have a higher incidence facial and body abnormalities.

### 4.3 Cardiovascular and renal system

There was a higher incidence of congenital heart anomalies in TS patients who had the complete loss of an X chromosome. Congenital heart disease has been recognized as one of the more common congenital malformations that are seen in patients with TS, affecting approximately half of patients (3, 8). The rate of heart color Doppler screening in our cohort was inadequate, with a screening rate of 53.89%. This led to a slightly lower rate of heart malformations than has been previously reported. This discrepancy may be due to differences in the means of detection (color Doppler) and the choice of age group. Two previous cohort studies that included the same detection method and age group had similar incidence rates of 19.6% and 25% (12, 13). Transthoracic echocardiography (TTE) with color Doppler is a useful screening tool to detect cardiovascular disease in infants and young children, but may be limited by its field of view and the availability of sonographic windows. Computed tomography (CT) and magnetic resonance imaging (MRI) were often used to supplement the anatomic and functional information that may not be evaluated with echocardiography, allowing for more cardiovascular abnormalities to be detected (18, 19). Given the limitations of the testing methods that were used to identify children with TS for inclusion in this study, not all children with TS and structural abnormalities may have been included. Congenital heart disease has been found to be more prevalent in individuals with a 45,X karyotype compared with those with X mosaicism or other X structural abnormalities (20).

Unlike cardiovascular disease, renal disease in children with TS has not been well studied (3). Tetsushi et al. reported a high prevalence of renal disease in TS patients (17%–42%) (21). Our study found that 21.27% of children with TS had renal disease, in particular renal pelvis separation (12.46%) and horseshoe kidney (7.29%). There are different views on the distribution of different genetic characteristics of renal disease with TS, with some studies suggesting that renal disease is more common in 45,X karyotypes (22–24) and others reporting no difference in between 45,X and X mosaicisms (21, 25, 26). Our Kruskal-Wallis analysis did not find a difference in the prevalence of renal disease between different X mosaicism proportions, with an overall p-value of 0.45.

### 4.4 Other comorbidities in children with TS

TS not only presents with physical and facial manifestations, cardiovascular, and renal disease, but with CNS, thyroid, gastrointestinal, and liver involvement as well. The present study identified a total of 160 children who had abnormalities of these systems, with no significant relationship with X mosaicism proportion (**Figure 2D**). As these symptoms involved about half of the children who were included in this cohort (47.90%), early detection is particularly important to permit adequate treatment and clear instructions should be given based on the latest guidelines for the management of children with TS. Prior work has shown that the incidence of some of these complications is related to age. For example, the prevalence of thyroid dysfunction and abdominal pain increase with age in TS patients (27, 28), and yearly liver function tests are also indicated (3). Overall, the complications in these organ systems were diverse and long-lasting, requiring comprehensive attention and treatment in clinical diagnosis. Future systematic analyses of specific clinical presentations may provide a more comprehensive understanding of the clinical presentations of Asian children with TS.

## 5 Limitations

There are several limitations to the present work. First, we included only those in whom FISH was applied to detect peripheral blood leukocytes and analyze the genetic distribution of sex chromosome number abnormalities among children with short stature. This did not include children with sex chromosome structural abnormalities seen on karyotyping. 32 cases of children in our statistics had structural abnormalities, the main karyotypes included were 6 cases of 45,XO/46,X,i(X)(q10), 6 cases of 46, X,i(X)(q10), 8 cases of 45,XO/46,X,+mar (8), as well as other abnormal karyotypes, such as 45,XO/46,X,i(X)(q15), 45,XO/46,X? idic(X)(q22), 45,XO/46,X,del(Y)(q12). Second, some of the comorbidities that were included in this study were not well detected. An example is otitis media, which had a prevalence of only 16.77%. Finally, we only analyzed the facial and body phenotypes of TS with X-chromosome number abnormalities, and did not set up a control group.

## 6 Conclusions

In conclusion, we found evidence of a relationship between the genetic distribution of TS and mosaicism. The average age of TS diagnosis was 9.2 years, with a higher number of children diagnosed at about 10 years and there is an increased rate of diagnosis around ages of 3 and 7. Abnormal face and body phenotypes and cardiovascular system abnormalities were more frequent in patients with a high proportion of X mosaicism or who had complete loss of an X-chromosome. Other comorbidities did not have this relationship. About future works, we will combine multidisciplinary diagnostics to investigate the specific clinical manifestations of TS, including the relationship between renal malformations and dysfunction and the relationship between ear disease and hearing loss.

## Data availability statement

The original contributions presented in the study are included in the article/**Supplementary Material**. Further inquiries can be directed to the corresponding authors.

## Ethics statement

The studies involving humans were approved by Ethics Committee of Henan Children’s Hospital. The studies were conducted in accordance with the local legislation and institutional requirements. Written informed consent for participation in this study was provided by the participants’ legal guardians/next of kin. Written informed consent was obtained from the individual(s), and minor(s)’ legal guardian/next of kin, for the publication of any potentially identifiable images or data included in this article.

## Author contributions

GW: Writing – review & editing, Conceptualization, Data curation, Formal analysis, Funding acquisition, Resources, Writing – original draft. XL: Data curation, Resources, Investigation, Writing – review & editing. MW: Writing – review & editing, Investigation, Methodology. JW: Data curation, Resources, Writing – review & editing. ZZ: Writing – review & editing, Data curation. KA: Writing – review & editing. DM: Resources, Writing – review & editing. YZ: Resources, Writing – review & editing. SL: Writing – review & editing, Data curation. YF: Writing – review & editing, Resources. DL: Resources, Writing – review & editing. YC: Resources, Writing – review & editing. HW: Methodology, Resources, Writing – review & editing.
