# Supplementary material for: Evaluating the relationship between the proportion of X-chromosome deletions and clinical manifestations in children with turner syndrome
Source: Front Endocrinol (Lausanne). 2024 Feb 28;15:1324160. doi: 10.3389/fendo.2024.1324160 (PMC10933015; doi:10.3389/fendo.2024.1324160)

Green represents the X chromosome centromere site

Red represents the Y chromosome centromere site

1．Sex chromosomes are XO

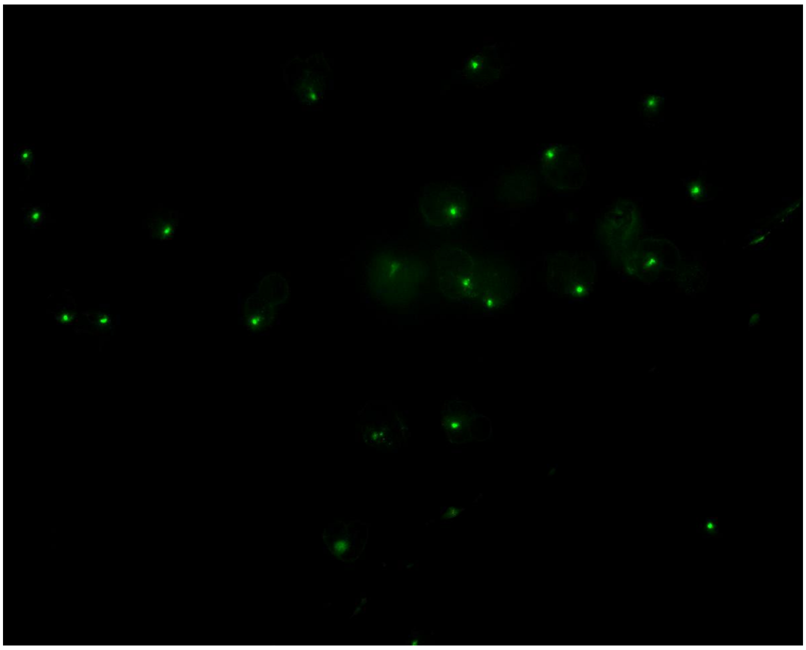

2．Sex chromosomes areX/XO

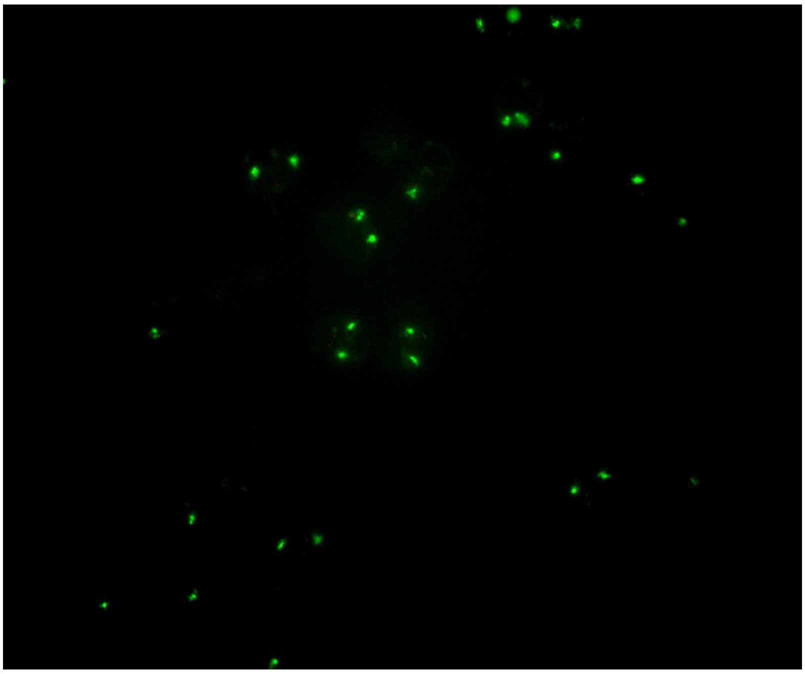

3. Sex chromosomes areX/XX/XXX

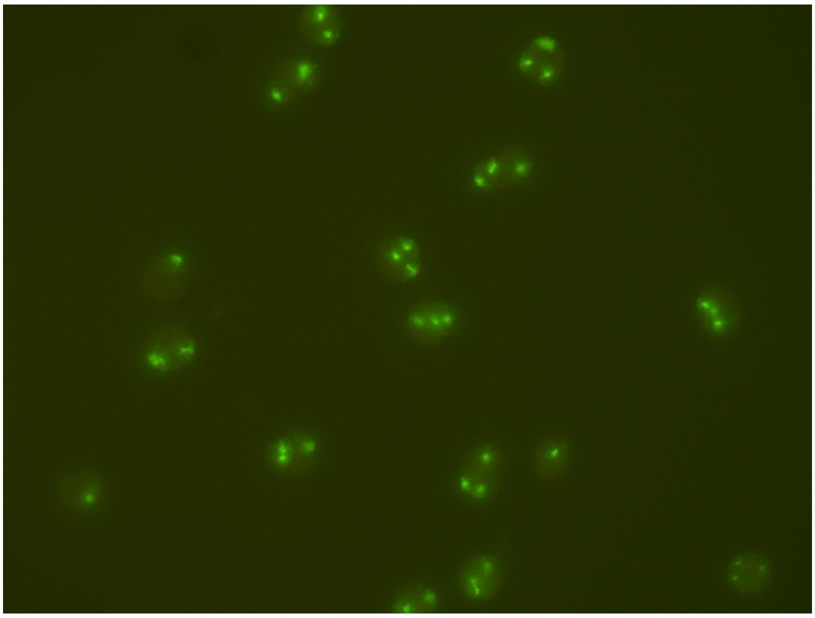

4. Sex chromosomes areX/XXX

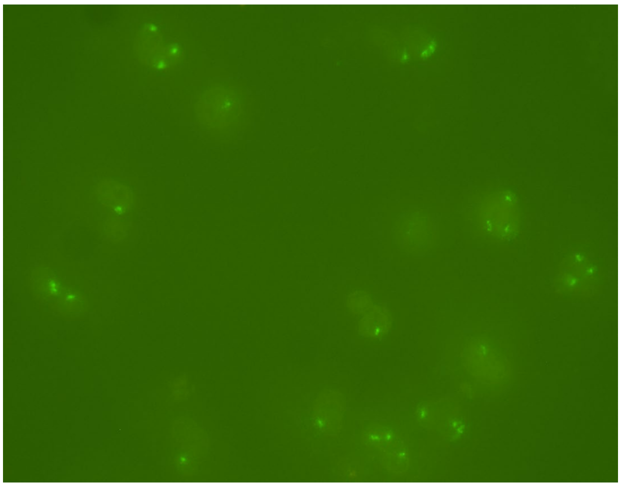

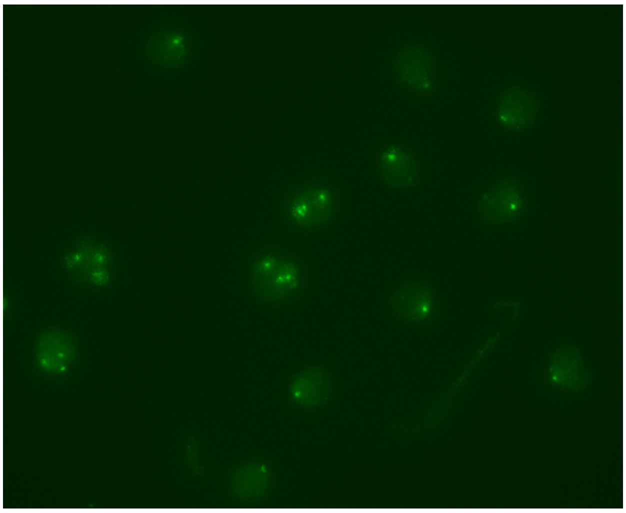

5. Sex chromosomes areX/XY

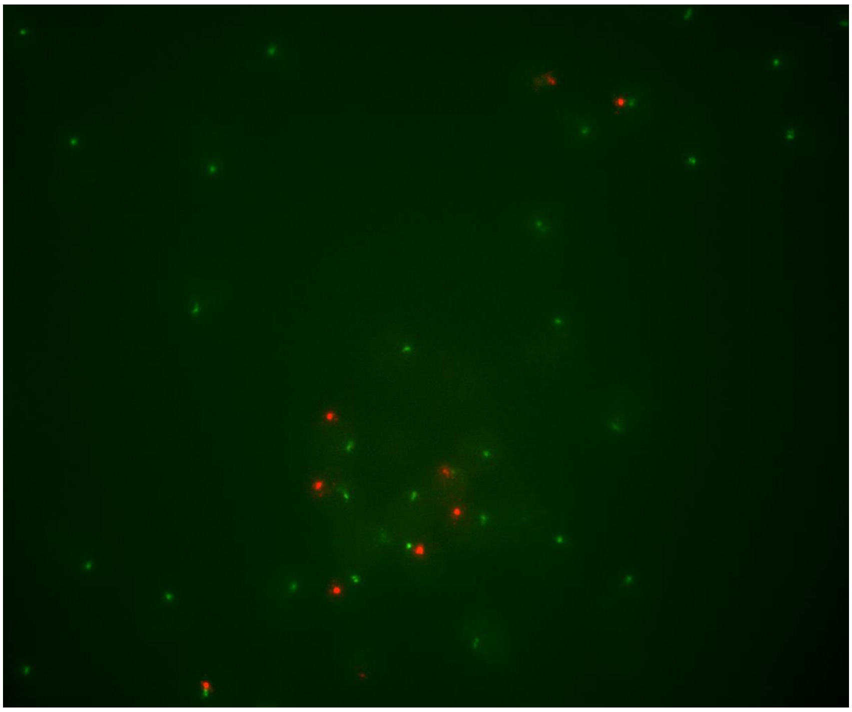

6. Sex chromosomes areX/XY/XYY

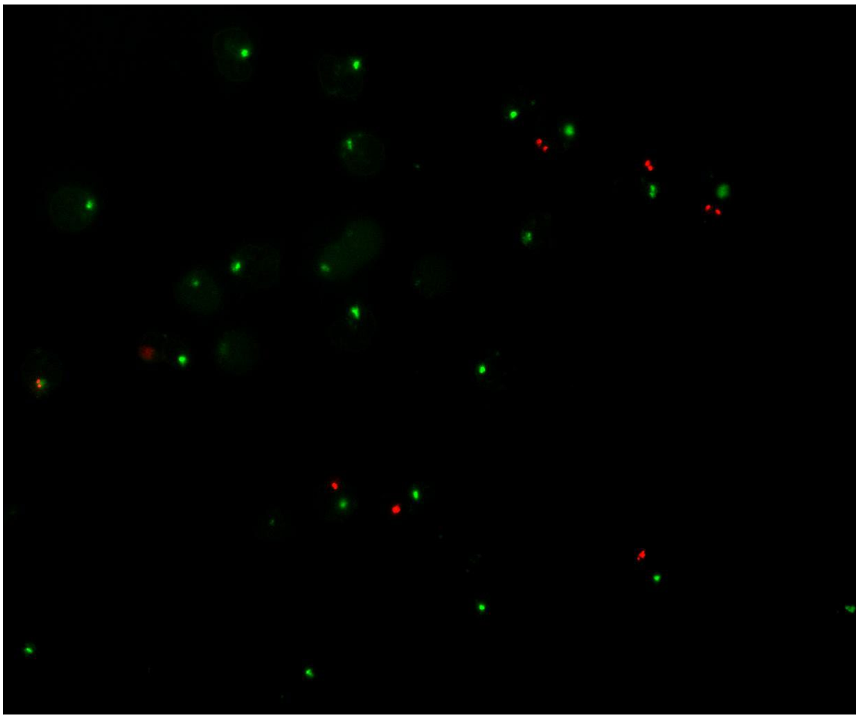

Supplement: Supplementary file 1 [file Image_1.pdf]
